# Correction to: *Hepatology, Medicine and Policy*: Articles with DOIs 10.1186/s41124-016-0014-8, 10.1186/s41124-016-0013-9 and 10.1186/s41124-016-0012-x

**DOI:** 10.1186/s41124-018-0042-7

**Published:** 2018-11-19

**Authors:** BioMed Central

**Affiliations:** https://www.biomedcentral.com/

## Correction

Following publication of these three articles [1–3], it was flagged that the articles have an incorrect issue copyright year of 2017, because of an xml-related error.

Please be advised, therefore, that the correct issue copyright year of these articles [1–3] is 2016.

The publisher apologizes for this processing error.
